# Age‐Independent Hypocholesterolemic Effects of Rice Protein via FXR‐Mediated Stimulation of Cholesterol Elimination

**DOI:** 10.1002/fsn3.70687

**Published:** 2025-07-28

**Authors:** Bingxiao Liu, Zhengxuan Wang, Mingcai Liang, Lin Yang

**Affiliations:** ^1^ School of Chemistry and Chemical Engineering Harbin Institute of Technology Harbin China

**Keywords:** bile acid excretion, cholesterol‐lowering, cholesterol output, FXR, growing and adult rats, rice protein

## Abstract

The present study investigated the effect of rice protein (RP) on plasma cholesterol clearance by administering diets containing either casein or RP to growing and adult male Wistar rats over a two‐week period. RP intake markedly upregulated the gene and protein expression of key regulators involved in cholesterol transport and metabolism, including ATP‐binding cassette transporter G1 (ABCG1), scavenger receptor class B type I (SR‐BI), ABCG5, bile salt export pump (BSEP), coactivator‐associated arginine methyltransferase 1 (CARM1), and farnesoid X receptor (FXR), in both age groups. These findings indicate that RP effectively promotes cholesterol and bile acid excretion. Consequently, RP significantly reduced cholesterol concentrations in both plasma and liver (*p* < 0.05), while fecal excretion of cholesterol and bile acids was significantly increased (*p* < 0.05). Overall, the results reveal a clear association between the cholesterol‐lowering effects of RP and its regulatory role in cholesterol elimination, irrespective of age. Notably, the ability of RP to stimulate the expression of ABCG1, low‐density lipoprotein receptor (LDLR), SR‐BI, ABCG5, BSEP, CARM1, and FXR remains unaffected by aging, suggesting a consistent hypocholesterolemic effect across different life stages.

## Graphical abstract

This study examined the impact of rice protein (RP) on cholesterol clearance using casein‐ and RP‐based diets in growing and adult male Wistar rats over 2 weeks. RP intake significantly upregulated key genes and proteins (ABCG1, SR‐BI, LDLR, ABCG5, BSEP, CARM1, FXR) involved in cholesterol transport and metabolism, thereby enhancing cholesterol and bile acid excretion. Consequently, RP reduced plasma and liver cholesterol levels while increasing fecal cholesterol and bile acid excretion, with consistent efficacy across different life stages.

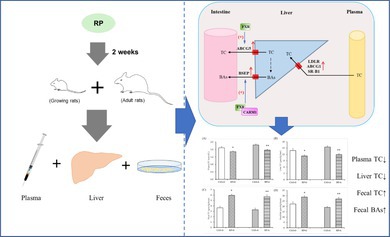

## 1 Introduction

Cholesterol level depends on cholesterol synthesis, cholesterol catabolism, cholesterol uptake, cholesterol output, and so forth. Elevated cholesterol levels in the blood are a significant risk factor for developing hypercholesterolemia, which can lead to conditions such as atherosclerosis and cardiovascular diseases. (Liu et al. 2021; Tufail et al. 2024; Xiao et al. 2024; Yang et al. 2019). Accordingly, it has contributed to a deeper understanding that the effective removal of cholesterol from plasma is the useful way to prevent hypercholesterolemia and is very beneficial for health.

Rice protein (RP), an important plant protein, has been shown to reduce the level of cholesterol in both the plasma and the liver, and therefore has a cholesterol‐lowering effect (Yang et al. 2007). Research has shown that the cholesterol‐lowering effect associated with RP is attributable to several factors, including its promotion of cholesterol breakdown into bile acids, reduction in cholesterol synthesis, and suppression of cholesterol absorption (Yang et al. 2011; Yang, Chen, et al. 2013; Yang, Han, et al. 2013). It has been demonstrated that the secretion of hepatic total cholesterol and very‐low‐density lipoprotein (VLDL)‐cholesterol into the circulation can be significantly depressed by RP. Conversely, biliary output of cholesterol and bile acids can be effectively stimulated by RP. These results underscore the potential of RP in regulating cholesterol output and reducing cholesterol levels (Yang and Kadowaki 2009). Unfortunately, the precise molecular mechanism through which RP regulates cholesterol efflux from the plasma remains to be fully clarified. This is despite the substantial evidence supporting its role in preventing hypercholesterolaemia.

The process of removing cholesterol from plasma can be considered complex, with several regulators having an impact on the outcome. ATP‐binding cassette transporter G1 (ABCG1) and scavenger receptor class B type I (SR‐BI) can mediate the transfer of cholesterol to the liver via plasma, responsible for the control of cholesterol efflux (Deminaa et al. 2016; Phillips 2014; Shen et al. 2018; Wang et al. 2004). ATP‐binding cassette transporter G5 (ABCG5) and ATP‐binding cassette transporter G8 (ABCG8) promote the secretion of hepatic cholesterol into bile, leading to the direct excretion of cholesterol to feces (Graf et al. 2003; Yu et al. 2014; Zein et al. 2019). Furthermore, the transformation of cholesterol into bile acids is a process that occurs within the liver. As a key metabolite of cholesterol, the elimination of bile acids via feces is considered an essential pathway for cholesterol removal from the body. The bile salt export pump (BSEP) serves as the primary transporter for the secretion of bile acids from hepatocytes into the biliary system, facilitating their excretion in feces and contributing to a reduction in cholesterol levels (Soroka and Boyer 2014; Dawson et al. 2009). In consideration of the aforementioned observations, it is evident that the efflux of cholesterol from the plasma, in conjunction with the excretion of both cholesterol and bile acids into feces, constitutes the primary pathways for the removal of cholesterol. This process is instrumental in engendering a cholesterol‐lowering effect (Alrefai1 and Gill 2007; Kosters and Karpen 2008; Phillips 2014). Thus, to explore the removal of the cholesterol mechanism involved with anti‐hypercholesterolemia, the expressions of ABCG1, SR‐BI, ABCG5/G8, and BSEP should be particularly taken into account.

The farnesoid X receptor (FXR) is a crucial metabolic nuclear receptor that plays a key role in cholesterol elimination by transcriptionally regulating ABCG5/G8 (Fitzgerald et al. 2002; Schmitz et al. 2001). It has been demonstrated by several studies that the expression of ABCG5/8 can be stimulated by FXR, thus increasing cholesterol excretion via hepatobiliary secretion (Graf et al. 2003; Yu et al. 2014; Zein et al. 2019). Furthermore, FXR is essential for regulating BSEP expression, thereby controlling the synthesis, transport, excretion, and reabsorption of bile acids. FXR activates BSEP to increase the transport and excretion of bile acids (Ananthanarayanan et al. 2001; Fu et al. 2022). Thus, FXR is regarded as playing a key role in regulating the excretion of cholesterol and bile acids; hence, it reduces plasma cholesterol level.

The process of cholesterol metabolism can be influenced by age, with the aging process being proposed as a significant risk factor for the onset of hypercholesterolaemia (Choi et al. 1989). However, cholesterol‐lowering effects induced by RP are observed both in growing and adult rats (Yang et al. 2007; Yang, Chen, et al. 2013; Yang, Han, et al. 2013). Thus, the question arises of why RP can reduce cholesterol levels in adult rats. To address this question and uncover the exact molecular mechanism through which RP regulates cholesterol elimination to exert its cholesterol‐lowering effect, both growing and adult rats were utilized in this study.

## 2 Materials and Methods

### 2.1 Animal Experiments

In alignment with our prior work, casein (CAS) and rice protein (RP) were selected as nutritional protein sources (Li et al. 2016, 2020; Liang et al. 2019; Yang, Chen, et al. 2013; Yang, Han, et al. 2013). The rice used in this study was *Oryza sativa* L. cv. *Longjing* 20, which was procured from the Rice Research Institute of Heilongjiang Academy of Agricultural Sciences in Jiamusi, China. Subsequently, RP was obtained using an alkaline extraction method. Casein, serving as the control protein, was sourced from Gansu Hualing Industrial Group in Gansu, China. Animal diets for growing and adult rats were developed according to the AIN‐93 guidelines established by the American Institute of Nutrition (Reeves et al. 1993).

For this experiment, growing male Wistar rats (body weights: 200–220 g) were provided *ad libitum* access to diets containing 20% dietary protein (as crude protein, CP) from either casein (CAS‐G) or RP (RP‐G), following the AIN‐93G guidelines, for a duration of 2 weeks. Similarly, adult male Wistar rats (body weights: 380–400 g) were fed diets containing 14% CP from casein (CAS‐A) or RP (RP‐A), based on AIN‐93M, for the same period. All rats were supplied by Vital River Laboratory Animal Technology Co. Ltd., based in Beijing, China.

The Harbin Institute of Technology Institutional Animal Care and Use Committee (IACUC‐2020009) approved all procedures. Each rat was housed individually in a metabolic cage within a controlled environment set at 22°C ± 2°C. The environment had 12 h of light (from 07:00 to 19:00) and 12 h of darkness (from 19:00 to 07:00 the next day). For acclimation, all rats were given unrestricted access to commercial pellets for the first 3 days. The commercial pellets were obtained from Vital River Laboratories in Beijing, China. Following this, the rats were randomly allocated to four groups (CAS‐G, RP‐G, CAS‐A and RP‐A), each comprising six similar‐weight animals.

Consistent with our prior studies, daily body weight and food intake were measured each morning prior to diet replenishment throughout the 2‐week feeding period. At the study conclusion, after a 12‐h fasting period, rats were euthanized under sodium pentobarbital anesthesia (50 mg/kg BW). Samples of blood were collected from the abdominal vein, immediately put on ice, and centrifuged at 12,000*g* for 5 min to obtain plasma, which was stored at −20°C until analyzed. Portions of the liver were rapidly frozen in liquid nitrogen and transferred to a freezer set at −80°C for storage until further analysis (Li et al. 2016, 2020; Liang et al. 2019; Yang, Chen, et al. 2013; Yang, Han, et al. 2013).

### 2.2 Measurement of Cholesterol and Bile Acids Contents

Commercial diagnostic kits (Nanjing Jiancheng Bioengineering Institute, Nanjing, China) were employed to quantify cholesterol concentrations in plasma, hepatic tissue, and fecal samples. Fecal bile acid levels were also determined using kits from the same supplier.

### 2.3 Quantitative Real‐Time PCR

After a 2‐week feeding period, TRIzol reagent kit (Invitrogen, Carlsbad, CA, USA) was used to isolate total RNA from the liver samples from both growing and adult rats according to the instructions of the manufacturer. Then, cDNA was synthesized from 1 μg of total RNA utilizing the PrimeScript 1st Strand cDNA Synthesis Kit (Takara Bio Inc., Otsu, Shiga, Japan). Quantitative real‐time PCR was carried out using the ABI 7500 sequence detection system (Applied Biosystems, Foster City, CA, USA) with SYBR Green (Takara Bio Inc., Otsu, Shiga, Japan). The mRNA levels of glyceraldehyde‐3‐phosphate dehydrogenase (GAPDH) were used as the reference for normalization purposes. The primer sequences are provided in Table 1. For comparison, the relative mRNA expression in the CAS‐G and CAS‐A groups was assigned a value of 1.00.

**TABLE 1:** Sequences of primers for quantitative real‐time PCR.

Gene	Forward	Reverse
GAPDH	ACAGCAACAGGGTGGTGGAC	TTTGAGGGTGCAGCGAACTT
ABCG1	CATGCTGCTGCCTCACCTCAC	CCGTCTGCCTTCATCCTTCTCTTG
ABCG5	AGCAGAAGTGGGACAGGAAA	CAGGAGGTAGGAGACGCAGT
BSEP	CCTGGCTCCGTCAAGTTCACATC	TTGGCTCAGAGGAGGCTACAGTAC
CARM1	TCAGTGAGCGAACAGAGGAGTCC	GTCCTTGAAGTCGGTGTGGTTCTG
FXR	ACATTCAACCATCACCACGCTGAG	TGCTGCTCTGAGGAGGACAAGG
SR‐BI	TCAGGGAGTTCAGACAAAAGGT	AGGACCAGGATGTTAGGCAGTA

### 2.4 Western Blotting Analysis

Hepatic proteins were extracted following the procedure detailed in our earlier research and subsequently used for western blot analysis (Li et al. 2016, 2020; Liang et al. 2019). The proteins were denatured by boiling for 5 min in 2× SDS sample loading buffer and then separated using 10% SDS‐PAGE. The gel was subsequently transferred onto a PVDF membrane (Millipore, Bedford, MA, USA). Afterward, the membranes were incubated with a blocking solution of 5% non‐fat milk in TBS at room temperature for 1 h. They were then subjected to overnight incubation at 4°C with primary antibodies targeting ABCG1 (Proteintech, Wuhan, China), ABCG5 (Proteintech, Wuhan, China), BSEP (Santa Cruz Biotechnology, Santa Cruz, CA, USA), coactivator‐associated arginine methyltransferase 1 (CARM1, Proteintech, Wuhan, China), FXR (Proteintech, Wuhan, China), SR‐BI (Proteintech, Wuhan, China), and β‐actin (Cell Signaling, Danvers, MA, USA). After three washes with TBST (TBS containing 0.1% Tween‐20), the membranes were incubated for 2 h at room temperature with secondary antibodies (Santa Cruz Biotechnology, Santa Cruz, CA, USA). Protein bands were detected using an ECL reagent (Beyotime, Shanghai, China), and band intensities were measured using QuantityOne software (Bio‐Rad, Hercules, CA, USA). Relative protein expressions in the CAS‐G and CAS‐A groups were set to 1.00 for comparison.

### 2.5 Statistical Analysis

The data are expressed as mean ± SEM. Statistical analyses were carried out using one‐way analysis of variance (ANOVA), with subsequent application of the least significant difference (LSD) test to compare group differences. A *p*‐value of less than 0.05 was considered statistically significant (Yang et al. 2007; Sharma and Kibria 2013; Juarros‐Basterretxea et al. 2024).

## 3 Results

### 3.1 Effect of Rice Protein on Plasma and Hepatic Cholesterol Levels

**FIGURE 1:**
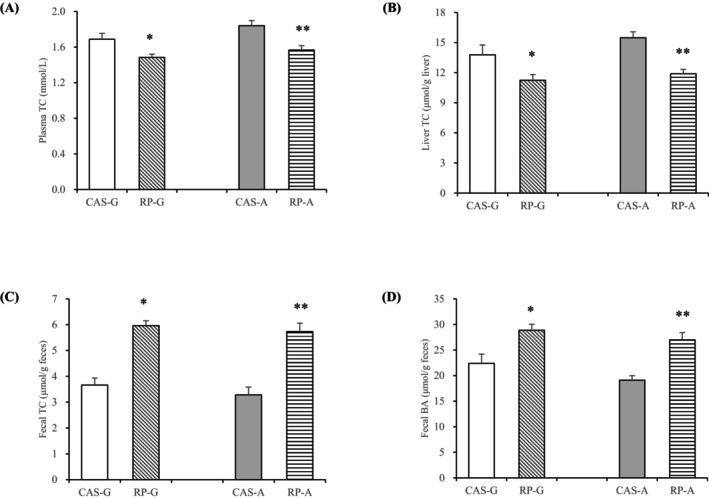
TC and BA levels in growing and adult male Wistar rats after 2 weeks feeding. (A) Plasma TC levels; (B) hepatic contents of TC; (C) fecal contents of TC; (D) fecal contents of BA. Values are the means ± SEM (*n* = 6). *p < 0.05, in comparison with CAS‐G. **p < 0.05, in comparison with CAS‐A. BA, bile acid; CAS‐A, adult rats fed with casein for 2 weeks; CAS‐G, growing rats fed with casein for 2 weeks; RP‐A, adult rats fed with rice protein for 2 weeks; RP‐G, growing rats fed with rice protein for 2 weeks; TC, total cholesterol.

As shown in Figure 1, after feeding for a 2‐week period, both RP‐G and RP‐A significantly lowered plasma cholesterol levels by 12.43% in growing rats and 14.67% in adult rats compared to CAS‐G and CAS‐A (*p* < 0.05, Figure 1A). Likewise, hepatic cholesterol levels were significantly reduced by RP‐G (18.36%) and RP‐A (23.19%) compared to CAS‐G and CAS‐A (*p* < 0.05, Figure 1B).

After a 2‐week feeding period, RP demonstrated cholesterol‐lowering effects, consistent with our earlier research (Yang et al. 2007; Yang, Chen, et al. 2013). The findings indicate that RP supplementation effectively reduces cholesterol concentrations in both growing and adult rats, irrespective of age.

### 3.2 Effect of Rice Protein on Fecal Excretion of Cholesterol and Bile Acids

After a 2‐week period of dietary feeding, RP‐G and RP‐A markedly enhanced fecal cholesterol excretion by 62.76% in growing rats and 74.63% in adult rats compared with CAS‐G and CAS‐A (*p* < 0.05, Figure 1C). Fecal bile acid excretion was also significantly increased, reaching 28.90% with RP‐G and 41.26% with RP‐A compared to CAS‐G and CAS‐A, respectively (*p* < 0.05, Figure 1D).

Given the observed reductions in plasma cholesterol levels with RP‐G and RP‐A, the finding that RP notably increased the fecal elimination of cholesterol and bile acids in both growing and adult rats further supports the notion that enhanced cholesterol excretion contributes to its cholesterol‐lowering effect, regardless of age.

### 3.3 Effect of Rice Protein on ABCG1 Expressions

In the current study, the cholesterol efflux mechanism exerted by RP was first investigated to elucidate the cholesterol‐lowering effect of RP.

**FIGURE 2:**
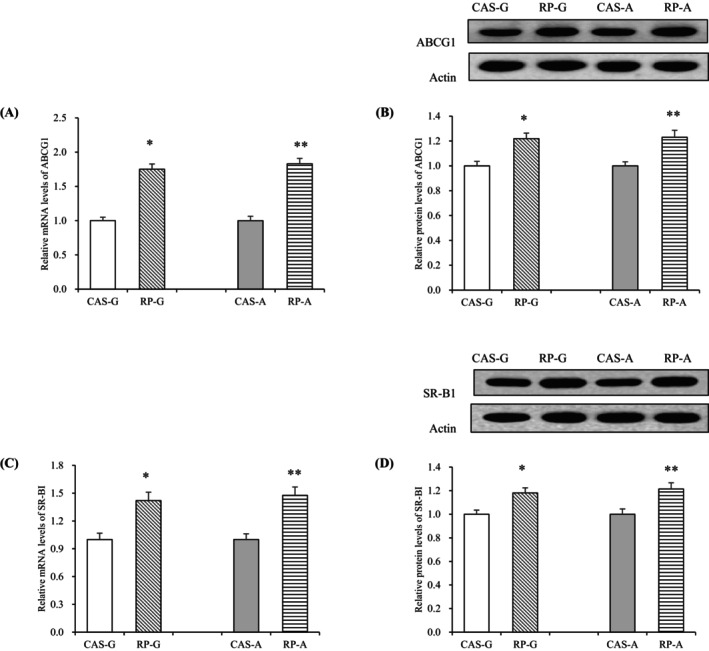
Hepatic mRNA levels and protein expressions in growing and adult rats. (A) Hepatic mRNA levels of ABCG1; (B) hepatic protein expressions of ABCG1; (C) hepatic mRNA levels of SR‐BI; (D) hepatic protein expressions of SR‐BI. Values are the means ± SEM (*n* = 6). *p < 0.05, in comparison with CAS‐G. **p < 0.05, in comparison with CAS‐A. ABCG1, ATP‐binding cassette transporter G1; CAS‐A, adult rats fed with casein for 2 weeks; CAS‐G, growing rats fed with casein for 2 weeks; RP‐A, adult rats fed with rice protein for 2 weeks; RP‐G, growing rats fed with rice protein for 2 weeks; SR‐BI, scavenger receptor class B type I.

As shown in Figure 2, RP significantly enhanced both protein and gene expression of ABCG1 in growing and adult rats after 2 weeks of feeding. Compared with CAS‐G and CAS‐A, RP‐G and RP‐A significantly upregulated ABCG1 mRNA levels (*p* < 0.05, Figure 2A). ABCG1 protein levels were markedly enhanced by 21.89% and 23.00% by RP‐G and RP‐A, respectively, compared to CAS‐G and CAS‐A (*p* < 0.05, Figure 2B), corresponding to a significant boost in gene expression. These findings indicate that RP consumption can upregulate ABCG1 expression, with no notable differences identified between RP‐G and RP‐A groups (*p* > 0.05), indicating that aging does not diminish the enhancement of ABCG1 expression induced by RP.

### 3.4 Effect of Rice Protein on SR‐B1 Expressions

SR‐BI has an essential function in cholesterol transport. In the current study, the impact of RP on SR‐BI expression was evaluated following a 2‐week feeding period.

Administration of RP led to significant elevations in SR‐BI mRNA levels in both RP‐G and RP‐A groups (*p* < 0.05, Figure 2C). Correspondingly, SR‐BI protein concentrations were notably higher in these groups, with increases of 18.07% for RP‐G and 21.45% for RP‐A, compared to CAS‐G and CAS‐A (*p* < 0.05, Figure 2D). These findings indicate that RP effectively modulates cholesterol efflux mechanisms, enhancing SR‐BI expression irrespective of age.

### 3.5 Effect of Rice Protein on ABCG5 Expressions

Consistent with the increased expression of ABCG1, hepatic mRNA and protein expressions of ABCG5 were also upregulated after RP supplementation.

**FIGURE 3:**
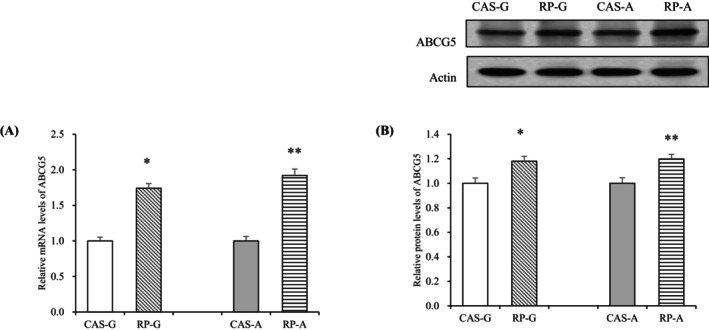
Hepatic mRNA levels and protein expressions of ABCG5 in growing and adult rats. (A) Hepatic mRNA levels of ABCG5; (B) Hepatic protein expressions of ABCG5. Values are the means ± SEM (*n* = 6). *p < 0.05, in comparison with CAS‐G. **p < 0.05, in comparison with CAS‐A. ABCG5, ATP‐binding cassette transporter G5; CAS‐A, adult rats fed with casein for 2 weeks; CAS‐G, growing rats fed with casein for 2 weeks; RP‐A, adult rats fed with rice protein for 2 weeks; RP‐G, growing rats fed with rice protein for 2 weeks.

Specifically, ABCG5 mRNA levels were markedly elevated in the RP‐G and RP‐A groups relative to the CAS‐G and CAS‐A (*p* < 0.05, Figure 3A). Correspondingly, ABCG5 protein expressions were markedly enhanced by RP‐G (18.07% increase) and RP‐A (19.83% increase) relative to CAS‐G and CAS‐A (*p* < 0.05, Figure 3B). These findings indicate that both RP‐G and RP‐A possess regulatory effects on cholesterol output regardless of age.

### 3.6 Effects of Rice Protein on BSEP and CARM1 Expressions

**FIGURE 4:**
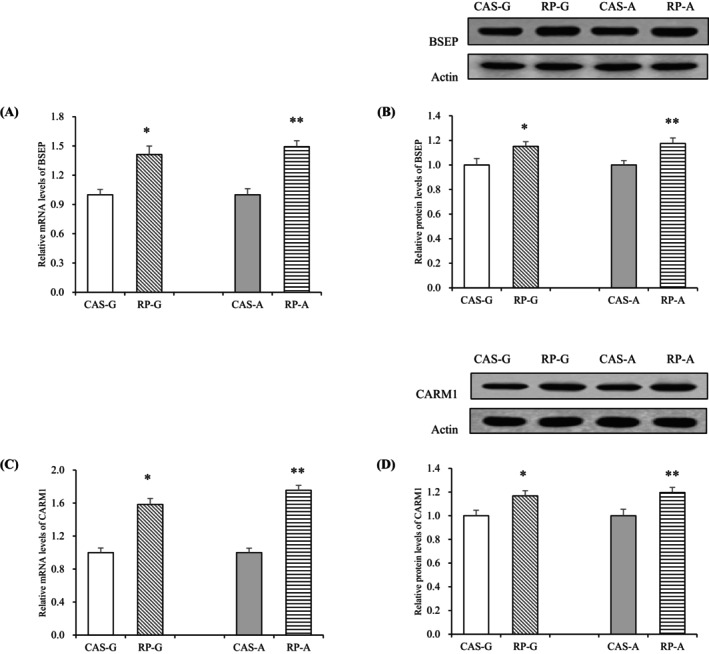
Hepatic mRNA levels and protein expressions in growing and adult rats. (A) Hepatic mRNA levels of BSEP; (B) hepatic protein expressions of BSEP; (C) hepatic mRNA levels of CARM1; (D) hepatic protein expressions of CARM1. Values are the means ± SEM (*n* = 6). *p < 0.05, in comparison with CAS‐G. **p < 0.05, in comparison with CAS‐A. BSEP, bile salt export pump; CARM1, coactivator‐associated arginine methyltransferase 1; CAS‐A, adult rats fed with casein for 2 weeks; CAS‐G, growing rats fed with casein for 2 weeks; RP‐A, adult rats fed with rice protein for 2 weeks; RP‐G, growing rats fed with rice protein for 2 weeks.

After a 2‐week feeding period, the mRNA expressions of BSEP (*p* < 0.05, Figure 4A) and CARM1 (*p* < 0.05, Figure 4C) were dramatically enhanced by the intake of RP.

In comparison to CAS‐G and CAS‐A groups, RP‐G administration resulted in a significant elevation of BSEP protein expression by 15.14%, while RP‐A increased it by 17.43% (*p* < 0.05, Figure 4B). Likewise, RP‐G and RP‐A treatments enhanced CARM1 protein levels by 16.72% and 19.61%, respectively, compared to CAS‐G and CAS‐A (*p* < 0.05, Figure 4D). These findings indicate that RP intake upregulates the expressions of BSEP and CARM1, suggesting a potential mechanism for cholesterol regulation.

Overall, both RP‐G and RP‐A clearly demonstrated the ability to enhance BSEP and CARM1 expression. This indicates that RP promotes bile acid excretion regardless of age.

### 3.7 Effect of Rice Protein on FXR Expressions

**FIGURE 5:**
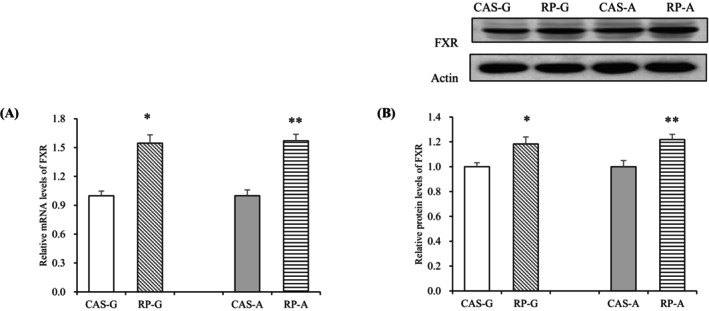
Hepatic mRNA levels and protein expressions of FXR in growing and adult rats. (A) Hepatic mRNA levels of FXR; (B) hepatic protein expressions of FXR. Values are the means ± SEM (*n* = 6). *p < 0.05, in comparison with CAS‐G. **p < 0.05, in comparison with CAS‐A. CAS‐A, adult rats fed with casein for 2 weeks; CAS‐G, growing rats fed with casein for 2 weeks; FXR, farnesoid X receptor; RP‐A, adult rats fed with rice protein for 2 weeks; RP‐G, growing rats fed with rice protein for 2 weeks.

As a key metabolic receptor involved in cholesterol and bile acid excretion, the mRNA expressions of FXR were strikingly elevated in the RP‐G and RP‐A groups compared to the CAS‐G and CAS‐A (*p* < 0.05, Figure 5A). Similarly, FXR protein expression was significantly elevated in the RP‐G group by 18.29% and in the RP‐A group by 21.87% when compared to the CAS‐G and CAS‐A (*p* < 0.05, Figure 5B). These results further suggested that RP could stimulate cholesterol output and bile acid excretion via activating FXR, despite the influence of aging.

## 4 Discussion

In order to thoroughly elucidate the hypocholesterolemic action of RP, the molecular mechanism by which RP regulates the removal of cholesterol was particularly emphasized in the current research. The findings demonstrated that RP's cholesterol‐lowering ability is associated with the regulation of ABCG1, SR‐BI, ABCG5, BSEP, CARM1, and FXR expression, all of which are critical for cholesterol excretion and bile acid elimination. Notably, this study is the first to reveal a direct association between the FXR‐BSEP pathway and the age‐independent cholesterol‐lowering effect of RP.

Cholesterol efflux is a primary output to eliminate cholesterol from plasma. The stimulation of cholesterol efflux to the liver is a major contributor to hypocholesterolemia (Phillips 2014). Cholesterol transfer to the liver through plasma is primarily facilitated by proteins such as ABCG1 and SR‐BI (Deminaa et al. 2016; Dergunov and Baserova 2022; Phillips 2014; Shen et al. 2018; Wang et al. 2004). Therefore, to investigate the mechanism of RP‐mediated cholesterol efflux, it is essential to examine the expression of ABCG1 and SR‐BI. Our results showed that RP significantly upregulated ABCG1 and SR‐BI expression, leading to decreased plasma cholesterol levels in both growing and adult rats. Furthermore, statistical analysis revealed strong negative correlations between plasma cholesterol levels and the expression of ABCG1 (*r* = −0.9005, *p* < 0.05) and SR‐BI (*r* = −0.8827, *p* < 0.05). These data suggest that the upregulation of ABCG1 and SR‐BI contributes to the cholesterol‐lowering effect of RP by enhancing cholesterol efflux. ABCG1 and SR‐BI participate in the transfer of cholesterol to high‐density lipoprotein (HDL), which involves the reverse cholesterol transport (RCT) (Deminaa et al. 2016; Phillips 2014; Shen et al. 2018; Wang et al. 2004). Thus, the current findings strongly support previous studies that RP could promote the RCT, which involves the HDL‐mediated transfer of cholesterol from peripheral cells (Li et al. 2014). In conclusion, these results provide convincing evidence that RP could act on the cholesterol‐lowering effect, which is associated with the stimulation of cholesterol efflux by RP.

After cholesterol is transported to the liver, ABCG5 facilitates the secretion of hepatic cholesterol into bile, which then moves into the intestine and is ultimately excreted in the feces (Graf et al. 2003; Yu et al. 2014; Zein et al. 2019). Thus, the biliary system serves as the primary pathway for eliminating hepatic cholesterol from the body, in which ABCG5 leads to the direct excretion of cholesterol to feces. In support of this view, the study examined a strong negative correlation between ABCG5 expression and hepatic cholesterol content (*r* = −0.8828, *p* < 0.05). Conversely, a robust positive correlation was observed between ABCG5 expression and fecal cholesterol excretion (*r* = 0.9058, *p* < 0.05). Some studies indicated that the ABCG5‐deficient mice exhibited increased plasma cholesterol accumulation (Graf et al. 2003; Li et al. 2019; Yu et al. 2014; Zein et al. 2019). In accordance with this view, RP significantly up‐regulated the expressions of ABCG5, resulting in the decreased plasma cholesterol levels in all RP groups. In this context, a significant negative correlation was identified between the expression of ABCG5 and plasma cholesterol levels (*r* = −0.8583, *p* < 0.05) in the study. Therefore, this study clearly demonstrates that the activation of ABCG5, which promotes the transfer of hepatic cholesterol into bile for fecal excretion, serves as a key mechanism underlying the cholesterol‐lowering effect of RP. This further suggests that ABCG5 plays a crucial role as the primary transporter involved in cholesterol elimination.

In addition to being excreted directly from the liver into feces, cholesterol is primarily excreted through the biliary pathway after being converted into bile acids. Hepatic cholesterol can be catabolized to bile acids, which are the main products of cholesterol catabolism. Thus, the transformation of cholesterol into bile acids serves as the primary pathway for its elimination through the hepatobiliary system, playing a key role in lowering plasma cholesterol levels. Studies have shown that RP can increase hepatic activity and gene expression of cholesterol 7α‐hydroxylase to stimulate bile acid synthesis (Yang, Chen, et al. 2013; Yang, Han, et al. 2013). As a consequence, RP enhances the conversion of cholesterol into bile acids, promoting its excretion into the bile rather than the circulation. This process significantly reduces both hepatic cholesterol content and plasma cholesterol content (Yang and Kadowaki 2009). The current findings validate and further extend this perspective. With a decreased hepatic content of cholesterol, fecal excretion of bile acids was respectively increased by RP in growing and adult rats. Results showed the significant negative correlation between bile acid elimination through feces and liver cholesterol content (*r* = −0.9115, *p* < 0.05), as well as plasma cholesterol level (*r* = −0.9184, *p* < 0.05), further demonstrating that the excretion of bile acids is one of the predominant mechanisms for cholesterol elimination exerted by RP. It is therefore clear that the cholesterol‐lowering effect of RP is due to its ability to improve the elimination of cholesterol by increasing the excretion of bile acids in the feces.

To clarify the molecular mechanism by which RP could stimulate bile acids excretion, the gene and protein expressions of BSEP, which could be pivotal in the circulation of bile acids, were investigated in this study. BSEP is a primary transporter for hepatic bile acid export and facilitates bile acids excretion to feces (Dawson et al. 2009; Soroka and Boyer 2014). It has been suggested that BSEP plays an essential role in regulating the rate‐limiting step of bile salt secretion in the liver (Alrefai1 and Gill 2007; Kosters and Karpen 2008). The results showed that there was a significant positive correlation between fecal bile acid excretion and the expression of BSEP (*r* = 0.9130, *p* < 0.05), indicating that BSEP may contribute to the enhanced bile acid excretion induced by RP. Some studies reported that the lack of BSEP could lead to the increase of cholesterol accumulation, resulting in hypercholesterolemia. In contrast, the stimulation of BSEP can enhance reverse cholesterol transport and promote bile acid excretion, ultimately contributing to cholesterol reduction (Alrefai1 and Gill 2007; Kosters and Karpen 2008; Soroka and Boyer 2014). In accordance with these facts, this study revealed a notable negative correlation between BSEP expression and both plasma cholesterol levels (*r* = −0.8736, *p* < 0.05) and hepatic cholesterol content (*r* = −0.8889, *p* < 0.05), respectively. Thus, it is evident that the stimulation of BSEP expression might be a mechanism by which RP can promote bile acids transport to reduce cholesterol level.

Cholesterol efflux and the elimination of cholesterol and bile acids are very important output pathways to eliminate cholesterol from plasma, in which ABCG5 and BSEP are the critical transporters for the removal of cholesterol and bile acids to feces. According to the extant literature, the FXR has been demonstrated to regulate ABCG5 and BSEP (Ananthanarayanan et al. 2001; Fitzgerald et al. 2002). As an important metabolic nuclear receptor, FXR significantly contributes to cholesterol and bile acid elimination by transcriptionally regulating ABCG5 and BSEP. Some studies have demonstrated that FXR enhances ABCG5 expression, resulting in the enhancement of cholesterol excretion through hepatobiliary secretion. In contrast, the inhibition of FXR could depress the expression of ABCG5 (Fitzgerald et al. 2002; Graf et al. 2003; Yu et al. 2014; Zein et al. 2019). In accordance with this view, we observed a strong positive correlation between the expression levels of FXR and ABCG5 (*r* = 0.9330, *p* < 0.05). As a fundamental regulator of bile acid metabolism, FXR is essential for controlling BSEP expression. It promotes BSEP expression, while its inactivation leads to a reduction in BSEP levels (Ananthanarayanan et al. 2001; Fu et al. 2022). In view of these facts, a strong positive association was identified between FXR expression and BSEP expression (*r* = 0.9072, *p* < 0.05), indicating that the FXR induced by RP up‐regulates the expression of BSEP. To be noted, the BSEP expression stimulated by FXR requires activation of CARM1 (Ananthanarayanan et al. 2004). In support of this perspective, our findings revealed strong positive correlations between CARM1 expression and both BSEP expression (*r* = 0.9372, *p* < 0.05) and FXR expression (*r* = 0.9288, *p* < 0.05). In addition, a strong positive correlation was identified between FXR expression and fecal bile acid excretion (*r* = 0.8609, *p* < 0.05) as well as fecal cholesterol excretion (*r* = 0.9211, *p* < 0.05), further supporting FXR's central regulatory role. More significantly, a strong inverse correlation was identified between FXR expression and plasma cholesterol concentration (*r* = −0.8736, *p* < 0.05). Consequently, after the intake of RP, the fact that the increased expressions of BSEP and ABCG5 via the stimulation of FXR could reduce cholesterol levels was identified in this study. Thus, this evidence revealed that the up‐regulation of FXR by RP might play an important role not only in stimulating cholesterol output to feces but also in promoting bile acid excretion in the feces, providing insight that the stimulation of FXR by RP might be a feasible mechanism for cholesterol removal to produce a hypocholesterolemic action.

In this study, it is important to highlight the impact of age on the regulatory role of RP in cholesterol elimination. It is well established that age can influence cholesterol metabolism, with aging being considered a key risk factor for the onset of hypercholesterolemia (Choi et al. 1989). It is important to highlight that under the current experimental conditions, with a hypocholesterolemic effect of RP in adult rats, the novel findings that the expressions of ABCG1, SR‐BI, ABCG5, BSEP, CARM1, and FXR were also upregulated by RP in adult rats emerged in this study, providing insight that the stimulation of cholesterol removal by RP cannot be attenuated by the aging process. As we all know, the biological efficiency of a protein largely relies on its amino acid composition (Cai et al. 2014; Yang et al. 2007; Yang, Chen, et al. 2013). Thus, although cholesterol transport regulation appears to be multifactorial, the amino acid composition of RP plays a crucial role and may be a key factor influencing this process. Several studies have shown that a higher arginine‐to‐lysine ratio in diets may reduce intestinal lipid absorption and enhance bile acid binding activity (Hu et al. 2017; Yang et al. 2012), suggesting that arginine plays a crucial role in regulating cholesterol metabolism. Given this, to explain the current phenomenon, the mechanism responsible for the influence of arginine on cholesterol removal was particularly taken into account. The data demonstrated strong positive associations between arginine consumption and the expression of ABCG1 (*r* = 0.8998, *p* < 0.05), SR‐BI (*r* = 0.8768, *p* < 0.05), ABCG5 (*r* = 0.9139, *p* < 0.05), BSEP (*r* = 0.8954, *p* < 0.05), CARM1 (*r* = 0.9087, *p* < 0.05) and FXR (*r* = 0.9119, *p* < 0.05). In view of these facts, a plausible explanation can be derived from the fact that the higher content of arginine in RP (87.8 μg/mg) than that in casein (33.3 μg/mg) might induce the stronger expressions of ABCG1, SR‐BI, ABCG5, BSEP, CARM1, and FXR in adult rats receiving RP‐A compared to those given CAS‐A. Further, a significant inverse relationship was observed between arginine consumption and plasma cholesterol levels (*r* = −0.8657, *p* < 0.05). Therefore, the fact that the aging process did not affect the removal of cholesterol in adult rats fed by RP‐A is convincing in this study, suggesting that the cholesterol removal mechanism exerted by RP through stimulating ABCG1, SR‐BI, ABCG5, BSEP, and CARM1 via activating FXR might be independent of the aging process. Undoubtedly, additional comprehensive studies are required to investigate the specific cholesterol transport mechanisms affected by RP in future studies.

## 5 Conclusion

This study elucidates the influence of RP on the expression of genes and proteins involved in cholesterol metabolism, such as ABCG1, SR‐BI, ABCG5, BSEP, CARM1, and FXR, in both growing and adult rats. The results offer strong evidence supporting the association between cholesterol‐lowering effects and the regulation of cholesterol elimination induced by RP. Notably, this research is the first to highlight that RP‐induced FXR activation may serve as a viable mechanism for enhancing cholesterol elimination, thereby exerting hypocholesterolemic effects. An intriguing observation is that the age of the rats does not diminish the cholesterol‐lowering efficacy of RP, suggesting that its impact on cholesterol regulation is robust across different life stages. This effect is likely attributed to the unique amino acid composition inherent in RP. However, further detailed studies are necessary to fully elucidate the precise mechanisms by which RP influences cholesterol metabolism to achieve its hypocholesterolemic effects.

## Author Contributions

Lin Yang: writing, conceptualization, supervision, project administration. Bingxiao Liu: investigation. Zhengxuan Wang: formal analysis, investigation. Mingcai Liang: formal analysis, investigation.

## Conflicts of Interest

The authors declare no conflicts of interest.

## Data Availability

Data are available on request.
